# Accounting for the phenomenology and varieties of auditory verbal hallucination within a predictive processing framework

**DOI:** 10.1016/j.concog.2014.09.002

**Published:** 2014-10-03

**Authors:** Sam Wilkinson

**Affiliations:** Department of Philosophy, Durham University, 50 Old Elvet, Durham DH1 3HN, UK

**Keywords:** Auditory-verbal hallucination, Psychosis, Schizophrenia, Predictive processing

## Abstract

Two challenges that face popular self-monitoring theories (SMTs) of auditory verbal hallucination (AVH) are that they cannot account for the auditory phenomenology of AVHs and that they cannot account for their variety. In this paper I show that both challenges can be met by adopting a predictive processing framework (PPF), and by viewing AVHs as arising from abnormalities in predictive processing. I show how, within the PPF, both the auditory phenomenology of AVHs, and three subtypes of AVH, can be accounted for.

## 0 Introduction

The positive symptoms of schizophrenia include delusions of control (“somebody else is controlling my actions”), thought insertion (“somebody is putting their thoughts into my head”) and auditory verbal hallucination (AVH) (hearing voices in the absence of a speaker). Perhaps the most popular theories for understanding these disparate symptoms are self-monitoring theories, which attempt to explain them as the product of one abnormality, namely, a problem with self-monitoring. According to these theories, our nervous systems distinguish self-generated from externally generated stimuli, through a process of self-monitoring. When this monitoring goes awry, self-generated stimuli are erroneously attributed to an external cause. The various positive symptoms all involve faulty monitoring and simply differ insofar as that which is failing to be properly monitored differs. In delusions of control it is bodily action, whereas in AVH and thought insertion it is widely thought to be inner speech (Feinberg, 1978; Frith, 1992; Jones & Fernyhough, 2007; Seal, Aleman, & McGuire, 2004). Although ingenious, the breadth of application of SMTs has recently been questioned by several theorists (Gallagher, 2004; Jones, 2010; Stephens & Graham, 2000; Wu, 2012). Such criticisms tend not to take issue with the application of SMTs to symptoms involving bodily action, like delusions of control and (their merely experiential analogue) illusions of passivity. Rather, they claim that SMTs struggle to account for AVH and thought insertion. In this paper, I will focus on AVH, and on two challenges in particular. They are:

*The Auditory Phenomenology Challenge* – How do you explain the auditory phenomenology of AVH if it is misattributed inner speech?

*The Varieties of AVH Challenge* – How do you account for the varieties of AVH if it is (always) misattributed inner speech?

In this paper, I suggest that both challenges can be met if we adopt a recently popular general framework for thinking about what the brain does (e.g. Clark, 2013; Friston, 2005, 2010; Hohwy, 2013) which we could call the predictive processing framework (PPF).

It is worth mentioning that the application of predictive processing to psychosis is not new. Indeed, Chris Frith, perhaps the best-known proponent of SMTs, has suggested something along these lines in Fletcher and Frith (2009). Since then, Adams, Shipp, and Friston (2013) have also suggested accounts of psychosis within the PPF. This work, however, does not focus on AVHs to the extent that I do, nor does it focus on the two challenges that I address here.

I proceed as follows. I start by presenting SMTs and show why they have been found attractive and plausible. I present the two challenges facing the application of SMTs to AVHs. I then introduce, motivate and clarify the PPF. I then present evidence suggesting that predictive processing might be disrupted in psychosis. Finally, I end by applying the PPF to voice-hearing, and show how it can, first, address the auditory phenomenology challenge, and second, nicely account for the three subtypes of AVH I present.

## 1 Self-monitoring theories

In this section I characterise SMTs, and describe the evidence that has been used to support them.

### 1.1 Introducing self-monitoring theories (SMTs)

Perhaps the first theorist to make use of self-monitoring was Helmholtz (1866). His concern, however, was not with psychopathology, but with the following problem presented by healthy visual cognition. When an image moves across the retina, how does our brain know whether it is the world moving across our eyes or our eyes moving across the world? Helmholtz suggested that our brain can tell the difference because when our eyes move there is a motor command. More specifically, information about the motor command, which Sperry (1950) later dubbed the “corollary discharge”, is used by the brain to predict the sensory consequences that would be produced by the eye movement. If the predicted and actual sensory consequences match then the brain infers that the change was self-generated and the conscious percept is adjusted accordingly. We can see exactly what happens when there is no such motor command, and hence no such adjustment, when we press on our eye with our finger. When we do this, the world itself seems to tilt and shake.

It took more than a hundred years for Helmholtz’s ideas to be applied to psychosis (Feinberg, 1978). Although Feinberg’s initial paper was on thought (which he took to involve ‘motor mechanisms’) and thought insertion, the easiest symptoms for which to introduce the account are delusions of control, since it is clear that, if anything involves motor commands, bodily actions do.^1^ In delusions of control, a subject may perform actions that are in keeping with her plans and intentions (for example, she might brush her hair), but she claims that somebody else is controlling her. Frith and Done (1989) took this to be a problem with self-monitoring. In particular, there is a mismatch between the predicted and actual sensory consequences of the bodily movement and so (as with Helmholtz’s ocular example) the movement is attributed to an external source. This, in principle, could be a problem with the generation of the prediction itself (e.g. the corollary discharge) or with the mechanism that compares expected and actual sensory (including proprioceptive) input, what became known as “the comparator”. Later manifestations of the self-monitoring theory (Frith, Blakemore, & Wolpert, 2000) saw comparator-based self-monitoring as the human body’s way of meeting a computational challenge, in particular, involving skilled reaching and online correction (see Wolpert, 1997 for a review of these computational approaches to motor control). This connection, in the late nineties, between the computational neuroscience of healthy cognition and the neuropsychology of schizophrenia undoubtedly contributed to the credibility of SMTs.

### 1.2 Support for SMTs

Whereas in Helmholtz’s example, the recognition by the nervous system that a certain stimulus is self-produced causes a correction of the conscious percept, in more typical bodily motor control, it results in sensory attenuation. The evolutionary benefit of this is clear enough: your nervous system needs to pay attention to stimuli that come from the outside, not the endogenous stimuli that (in a well-functioning system) will be harmless and irrelevant. Various data suggest that something goes wrong with this monitoring and subsequent attenuation (Blakemore, Smith, Steel, Johnstone & Frith, 2000). The most striking such datum is the reported finding that subjects with diagnoses of schizophrenia can tickle themselves. The postulated explanation for this is that there is a mismatch between expected and actual sensory consequences and the sensory consequences are not attenuated: the tickling sensation is like being tickled by somebody else. Typical subjects can’t tickle themselves because their nervous systems accurately monitor, and successfully attenuate, the sensory consequences of the tickling movements (Blakemore, Wolpert & Frith, 1999).

It is not only with bodily action that support has been shown for the claim that self-monitoring goes awry in schizophrenia. Studies showed that patients with diagnoses of schizophrenia are more likely to misattribute their own voices than healthy controls (Cahill, 1996; Johns et al., 2001). For example, Johns et al. (2001) got subjects to read words aloud and played them feedback of their voices with mild acoustic distortions. The subjects with diagnoses of schizophrenia were considerably more likely than healthy controls to claim that the voice they heard was someone else’s. Indeed, support that this was directly related to the production of hallucinations was supported by the result that, within the “schizophrenia” group, those who experienced hallucinations were more likely to misattribute their voice than those who did not hear voices.

### 1.3 Applying SMTs to AVH

Several theorists (Feinberg, 1978; Frith, 1992; Jones & Fernyhough, 2007; Seal et al., 2004) have attempted to explain AVHs in terms of inner speech misattribution, based on self-monitoring abnormalities. Although from a pre-theoretical standpoint it is not obvious that inner speech involves motoric elements, this has been empirically supported by several electromyographical (EMG) studies (which measured muscular activity during inner speech) some of which date as far back as the early 30s (e.g. Jacobsen, 1931). Later experiments made the connection between inner speech and AVH, showing that similar muscular activation is involved in healthy inner speech and AVH (Gould, 1948; McGuigan, 1966). The involvement of motoric elements in both inner speech and in AVH is further supported by findings from Gould (1950), who showed that when his subjects hallucinated, subvocalisations occurred which could be picked up with a throat microphone. That these subvocalisations were causally responsible for the inner speech allegedly implicated in AVHs, and not just echoing it (as has been hypothesised to happen in some cases of verbal comprehension (cf. e.g. Watkins, Strafella, & Paus, 2003)), was suggested by Bick and Kinsbourne (1987), who demonstrated that if people experiencing hallucinations opened their mouths wide, stopping vocalisations, then the majority of AVHs stopped.

To recap, SMTs have been presented as a unifying model for understanding the positive symptoms of schizophrenia. The idea is that there is one deficit concerning the monitoring of self-produced stimuli, and the symptoms differ because there are different kinds of self-produced stimuli. In delusions of control it is physical action, whereas in AVH and thought insertion it is inner speech.

## 2 Two challenges facing the application of SMTs to AVH

As mentioned, criticisms of SMTs tend not to take issue with the role of self-monitoring deficits in delusions of control, but rather concern the aforementioned extension of self-monitoring to symptoms that don’t involve bodily action, namely, AVH and thought insertion. In this paper, I specifically focus on two challenges facing the application of SMTs to AVHs.

### 2.1 The Auditory Phenomenology Challenge

How are we to account for the distinctly *auditory* phenomenology of certain AVHs? As Cho and Wu (2013) put it, if a theory claims that AVHs are misattributed inner speech, it must explain how we get a transformation from

the experience of the subject’s own inner voice […] often lacking acoustical properties such as pitch, timbre, and intensity into the experience of someone else’s voice with acoustical properties. (p.2)

As Wu (2012) puts it in an earlier paper, “we must explain this ‘transformation’ from the normal to the pathological” (p.94). Although one can question the premise that inner speech lacks auditory phenomenology (McCarthy-Jones and Fernyhough, 2011; Moseley & Wilkinson, 2014), even granting that this is the case, the PPF makes this ‘transformation’ less perplexing.^2^

### 2.2 The Varieties of AVHs Challenge

Jones (2010) emphasises the heterogeneity of AVHs:

The term AVH encapsulates a diverse phenomenological experience, which may involve single and/or multiple voices, who may be known and/or unknown, speaking sequentially and/or simultaneously, in the first, second, and/or third person and which may givecommands, comments, insults, or encouragement. (2010, p.566)

In particular, Jones presents two models for understanding AVHs and argues that both are promising for understanding different subtypes of AVH.

The first model he examines is the “memory-based” model proposed by Waters, Badcock, Michie, and Maybery (2006), which he suggests fits well with the phenomenological features of AVHs that appear to be the upshot of a traumatic experience. In these AVHs you often find, for example, instructions to self-harm in a voice that is recognisably that of the abuser. Even more strikingly, the contents of these AVHs “can be related to what was said during or surrounding these events (e.g. if you tell anyone I’ll kill you)” (Jones, 2010, p.568). However, these form only a relatively small subset of AVHs (e.g. 4 out of 24 in Fowler et al., 2006). A larger subset of AVHs seem to serve the function of regulating or commenting on current events (e.g. Nayani and David (1996) reported that for 46% of their sample, the voice had replaced their “voice of conscience”). For these, Jones (2010) suggests that his second candidate model is a better option. This model is precisely the inner-speech-based SMTs that we introduced in Section 1.

The moral of this is that it is very difficult to account for all this variety within a model that explains AVHs in terms of misattributed inner speech. I am in complete agreement with Jones that we need different models for different subtypes.^3^ I would simply add to Jones’s suggestion, however, that we distinguish frameworks, theories, and models.

Roughly, the distinction is as follows. *Frameworks* are very broad; they are ways of approaching a particular domain of inquiry (e.g. the brain and cognition). It is *within* them, whether implicitly or explicitly, that theories are built.^4^ *Theories* are falsifiable claims (rather than ways of approaching something) at a high level of generality that explain a phenomenon or class of phenomena by elucidating the fundamental nature of the phenomenon, or by postulating certain rules or principles that govern or define the phenomenon (e.g. a self-monitoring theory of schizophrenia). *Models* are at a lower level of generality; they explain how a particular kind of phenomenon arises, sometimes in terms of other, hopefully better understood, phenomena (e.g. a misattributed inner speech model of AVH). Theories might suggest candidate models. So a self-monitoring theory of the primary symptoms of schizophrenia might suggest a misattributed inner speech model for AVHs occurring in the context of schizophrenia. Models should be applicable to individual cases. Thus, given a theory that posits one kind of deficit, different models could well be needed for different symptomatic manifestations of that underlying deficit.

In a nutshell, I will suggest that the different subtypes need (as Jones, 2010 suggests) to be explained in terms of different aetiological models. However, these models are to be built within the same framework, the predictive processing framework that I will present now.

## 3 The predictive processing framework (PPF)

I’d like to put psychosis to one side, and focus, in this section, on presenting a framework for understanding the brain and cognition *generally*. We will later see how this might apply to AVH. I will start with an initial presentation of the PPF, and then present some conscious effects that are suggestive that the brain is engaged in predictive processing.

### 3.1 An initial presentation: “inference” and efficiency

According to the PPF, the brain’s main task is to “infer” from incoming signals, what the causes of those signals are, in other words, settle on a hypothesis about what is “going on”.^5^ However, the incoming signal, namely, the proximal stimulus on sensory receptors, underdetermines distal causes. Since inputs are noisy and ambiguous, there is no one-to-one mapping: the same stimulation can be brought about by two very different distal causes (and different stimulation in different circumstances can be caused by the same distal cause). Given that more than one hypothesis is compatible with the incoming signal, how does the brain settle on one hypothesis rather than another? It needs to take two things into account: first, the fit of the input with the hypothesis, and, second, how statistically likely that hypothesis is (the “prior probability”), at least as far as the brain is concerned (this is *subjective* rather than objective probability). A hypothesis could fit the input extremely well, but its prior probability could be so low that it isn’t even considered. Conversely, a hypothesis could have such a high prior probability, that, even though it doesn’t fit the input well, it is settled upon.

Although this is a picture of what the brain has to do in order to do a good job of “inferring” what is going on, it is abstracted from the temporal dynamics of the brain’s functioning. In reality, the inputs, hypotheses and prior probabilities (priors) are in constant hierarchical interaction with each other. This is where the notion of prediction comes in. What the selection of a hypothesis does is that it determines a set of predictions about subsequent inputs, namely, inputs that are compatible with the hypothesis. If the hypothesis does a good job of predicting inputs, it will be kept. If it does a bad job, it will be tweaked or abandoned altogether in favour of another hypothesis. In other words, one hypothesis is selected rather than another if it better *minimises prediction error*.

Some theorists (e.g. Hohwy, 2013) sum up the PPF by saying, for example, that the brain is in the business of minimising prediction error.^6^ This prediction error minimisation is not only taken to account for perception and cognition, but for action as well (see e.g. Adams et al., 2013). Instead of there being motor commands, as on the standard picture, what you have are predictions, which are then fulfilled by the subsequent bodily movement, thereby also being a case of prediction error minimisation. This is often called “active inference” (Friston, 2009), which Pickering and Clark (2014) helpfully gloss as follows: “the combined mechanism by which perceptual and motor systems conspire to reduce prediction error using the twin strategies of altering predictions to fit the world and altering the world (including the body) to fit the predictions” (p.1).

This picture has interesting consequences for how we are to view the role of input on sensory receptors and its impact on higher cortical regions. According to the PPF, the only information that gets passed on up the hierarchy is *prediction error*. Indeed, as some recent theorists nicely put it:

an expected event does not need to be explicitly represented or communicated to higher cortical areas which have processed all of its relevant features prior to its occurrence.

[Bubic, von Cramon, and Schubotz (2010, p.10)]

This stands in sharp contrast to the standard (and admittedly intuitive) view of perception (what might be called a “bottom-up feature-detection view”). On such a view, inputs come in, are processed, and passed on. On the PPF, only the prediction error gets passed on, and therefore the vast majority of what determines your perceptual experience is what your brain has already predicted, your brain’s best hypothesis. In short, on the PPF, the role of the incoming signal in determining conscious perception is much less great than on standard bottom-up views.

### 3.2 Precision weighting, second-order prediction and “attention”

Although incoming signals are ambiguous, in different contexts, the degree of ambiguity will differ. To maximise its predictions, the brain needs to accurately estimate how much ambiguity (uncertainty) there will be. In contexts where low ambiguity is expected, higher precision will be demanded, and *vice versa*. This is called “precision weighting” in the literature (Feldman & Friston, 2010; Friston, 2009; Hohwy, 2012), and is taken to be modulated by neurotransmitters such as dopamine (Corlett, Taylor, Wang, Fletcher, & Krystal, 2010).^7^

Recently, theorists (Feldman & Friston, 2010; Hohwy, 2012) have equated this precision weighting with what we often call “attention”. As is well known, attention can be brought about endogenously or exogenously, namely, either because the subject is in a state of wanting to attend to something (endogenous attention), or because something in the environment attracts attention (exogenous attention).

Suppose I’m talking to someone in a crowded bar. By using information from seeing the person’s lips move, my brain has certain expectations about what sounds are likely to be produced. That part of my visual field, and the corresponding part of my “auditory field”, now has relatively high precision attributed to it, largely because I am interested in what’s going to be said (endogenous focal attention). Everything else around that, both visual and auditory, has relatively low precision. My brain has a very gist-like hypothesis about what is going on there, and, any prediction error regarding that has low weighting. Of course, a loud peripheral bang or camera flash can grab my attention (a case of exogenous focal attention). That would constitute a strong enough input to overcome whatever down-modulation my brain has placed on that peripheral prediction error.

To sum up, then, we can think about the directing of attention as “turning up” the expected precision of an incoming signal, which amounts to turning up the “gain” on any prediction error. This can be viewed normatively. That is to say, not only can your brain get its predictions wrong, it can also get the expected precision of its predictions wrong, i.e. its second-order predictions. Your brain can wrongly think that it is in a more or less ambiguous environment than it actually is in. And this will mean too much or too little weighting on prediction error.

### 3.3 Conscious effects of predictive processing

#### 3.3.1 Binocular rivalry

This is perhaps the most common example used in support of the PPF. It speaks strongly against a bottom-up view in favour of a more predictive or “inferential” view. Under experimental conditions, each eye is presented with a different, but meaningful, stimulus. One standard example involves presenting one eye with a picture of a house, and the other with a picture of a face. Subjects do not report visually experiencing, as one might expect, a mixture of face and house. Rather, they experience a “bi-stable” switching, from face to house, and back, and so on (the switching is often reported as a gradual “breaking through” of the other image). As Hohwy, Roepstorff, and Friston (2008) point out, this can be nicely explained within the PPF, as a reasonable response to highly un-ecological circumstances. In a nutshell, you experience the bi-stable state because your brain is switching between hypotheses about what is out there. When it settles on one hypothesis, say, the face hypothesis, inputs from the house image fail to accord with this hypothesis and prediction error is sent up the hierarchy. When enough prediction error accumulates, the hypothesis switches (and with that, what the subject consciously experiences) to the house hypothesis, but then the input from the face image doesn’t accord, and so on, and so forth. The fact that this bi-stable switching only occurs with certain well-chosen stimuli, suggests the operation of what might be called a “hyperprior”: an expectation about the world that is stable, and often at a high degree of abstraction. The “hyperprior” in this case is that faces and houses, being at different scales, cannot occupy the same region of the visual field at the same time. As a result, the “face-house” hypothesis about what is out there never presents itself.

Note how binocular rivalry puts pressure on a “bottom-up” view of perceptual experience. As Hohwy (2013) puts it:

During rivalry, the physical stimulus in the world stays the same and yet perception alternates, so the stimulus itself cannot be what drives perception (p.20)

One might think that binocular rivalry could result from the operation of a different principle, and one that is compatible with a largely “bottom-up” view, namely the principle: “If your eyes each have coherent but incompatible inputs, don’t receive input from both at once.” This nicely illustrates how predictive processing concretely differs from a bottom-up view such as this one. When the subject experiences a house, on the bottom-up eye-selection hypothesis, this means that the eye being presented a house is passing on a signal, whilst the eye being presented a face is not. On the predictive processing view, it is the opposite: the eye being presented the face is passing on a signal, namely a prediction error signal.

One reason for rejecting the eye-selection account is that it doesn’t (unlike the build-up of prediction error) explain the stable switching back-and-forth. A second, more striking, reason is that this account was falsified by Diaz-Caneja (1928) who discovered that if each stimulus picture is cut in half and swapped over, so that each eye is being presented with a half-face half-house image (split down the middle) there is the same experience as before, namely, a rivalry between a complete percept of a face and a house.

#### 3.3.2 The Hollow Mask Illusion

When you are presented with a rotating mask that is slowly turned to present you with the concave back of the mask, your brain “corrects” the concave stimulus into a convex stimulus. (You yourselves can experience this illusion on several sites on the internet.) You experience the concave back of the mask, as convex. Again, this is due to a very strong prior (perhaps worthy of being called a hyperprior), that over-rides the incoming signal. The prior in question is that the faces you will encounter will always be convex (a fair expectation!). This prior is so strong that even though the “concave face” hypothesis would better match the input, it is never selected. Given the highly specialised processing that faces receive in human cognition, this is perhaps hardly surprising.

#### 3.3.3 The McGurk Effect

Moving away now from uni-modal priors, there is an effect that deals with highly flexible, concrete, cross-modal priors. This is the fact that what you see will affect your brain’s expectations about what it hears. If a subject is played a video of someone appearing to say “ga, ga”, but you play them a synchronous audio track of someone saying “ba, ba”, you aurally experience “da, da” (something “in between”). This is called the McGurk Effect. The visual input changes your brain’s expectation concerning the auditory input, and interprets it differently.

We can see the same cross-modal predictive processing (vision having an effect on audition) at work in more everyday cases. If you look across a crowded bar at someone ordering a drink, by looking at her lips, you can actually *hear* her order. Your brain uses the visual input to inform its priors about what is being said. If you had tried to hear her order with your eyes closed, there is no way you would have been able to single out her voice from the noise of the crowded room. Your brain just wouldn’t have had the visual cues to inform its expectations.^8^ Furthermore, since what she says is what matters to you at that moment, you “turn up the gain” on prediction error that is relevant to hypotheses concerning what she says.

### 3.4 Clearing up potential confusion: what the brain does and what the person does

Although this predictive processing is extremely relevant for our conscious experience, we need to be very careful to distinguish what our brains do from what we do. One nice way of seeing this distinction is by distinguishing “surprisal” (Tribus, 1961) and “surprise”, where the former is what is surprising to your brain (as signalled by prediction error), and the latter is what is surprising to you.^9^ These two come apart. If you look out of your window and see an elephant on the lawn, you might be very surprised. However, the fact that you see straight away that it is an elephant shows that your brain has already minimised the prediction error and settled on the elephant-on-lawn hypothesis. Conversely, when you are attempting a binocular rivalry task, and you’ve done it before, the switching doesn’t surprise you, however, your brain is switching between hypotheses precisely because it is struggling to keep “surprisal” to a sufficiently low level. Since we are concerned with AVHs, a conscious phenomenon, it is vital to understand this relationship between what the brain does, and what the person does, and, analogously, predictive processing on the one hand, and conscious experience on the other. Roughly, your conscious percept is determined by the overall hypothesis that your brain has adopted in order to minimise prediction error.

## 4 Predictive processing and psychosis

Now that I have presented the PPF, we can see what relevance it has for psychosis in general, and for AVH in particular. Two sources of evidence that support the hypothesis that predictive processing abnormalities might be implicated in psychosis are, first, that the conscious effects that have been taken to be suggestive of predictive processing are altered in subjects with diagnoses of schizophrenia, and, second, that there are behavioural results from eye tracking that are nicely explicable in terms of predictive processing.

### 4.1 Conscious effects are altered in patients with diagnoses of schizophrenia

Suppose that psychosis can be understood, in part, at least, as a breakdown in normal predictive processing. And suppose that, as we have suggested, the conscious effects just mentioned are the product of normal predictive processing. One would expect patients with schizophrenia diagnoses to experience these effects differently from the normal population. This is exactly what the evidence suggests.

Schneider, Leweke, Sternemann, Weber, and Emrich (1996), Schneider et al. (2002) and Emrich, Leweke, and Schneider (1997) demonstrate that subjects with schizophrenia diagnoses do not experience the Hollow Mask Illusion. They see the mask, correctly, as hollow. Their brain does not “correct” the input.

Pearl et al. (2009) showed that patients with diagnoses of schizophrenia experience the McGurk Effect less. In other words, the visual stimulus corrects the auditory input less, or not at all. Pearl et al. (2009) put this in terms of a “decreased reliance” on visual cues. Results with similar implications are reported by Ross et al. (2007) with regards to visual enhancement of speech comprehension in noisy environmental conditions. In other words, subjects with diagnoses of schizophrenia showed an impairment in their ability to enhance their auditory perception with visual cues. Within a PPF this would be put in terms of either decreased efficacy of auditory priors informed by the visual information, or in terms of excessively weighted bottom-up prediction error (or both).

As for binocular rivalry, Heslop (2012) showed that switching rates in binocular rivalry were, on average, significantly slower in subjects with schizophrenia (0.28 switches per second, versus 0.54 in the non-clinical population).

### 4.2 Evidence from eye tracking

Following Adams, Perrinet, and Friston (2012), I distinguish three eye-tracking tasks where the performance of patients with diagnoses of schizophrenia differs significantly from healthy controls.

First, there are cases of impaired tracking during visual occlusion (Hong, Avila, & Thaker, 2005; Thaker et al., 1998). In other words, when a moving target is temporarily occluded from view, controls are much better than subjects with diagnoses of schizophrenia at keeping track of the target when it comes back into view.

Second, patients with diagnoses of schizophrenia show impaired “repetition learning.” When a target trajectory is repeated, healthy controls achieve optimal performance whereas subjects with schizophrenia diagnoses do not (Avila, Hong, Moates, Turano, & Thaker, 2006).

Thirdly, there are cases of what, in the literature, is called “paradoxical improvement”, namely, where patients with a supposed illness or deficit perform better at a particular task than healthy controls (the fact that patients with schizophrenia do not experience the Hollow Mask Illusion might be thought of as a “paradoxical improvement” since their percept is more veridical). The task in question involves keeping track of a target that rapidly and unexpectedly changes direction. Subjects with schizophrenia are better at keeping track of the target than healthy controls (but only very shortly after the change in direction).

Working within the PPF, Adams et al. (2012) explain these three discrepancies in terms of differing reliance on predictions (priors) and prediction error respectively. The first two tasks are improved by reliance on prediction, whereas the third task, being deliberately unpredictable, is hindered by prediction, and would be improved by a higher weighting on the prediction error. Note, however, that, like the Hollow Mask Illusion, the “paradoxical” improvement is shown on a stimulus that is statistically un-ecological. Just as we encounter convex faces and never concave ones, the world is full of statistical regularities and it is more adaptive to be able to exploit those regularities efficiently than to slightly outperform others in the rare instances when stimuli are totally unpredictable.

## 5 Addressing the two challenges

I will start by showing how the PPF addresses the *Auditory Phenomenology Challenge*. In particular, I will show how it circumvents the issue of explaining the “transformation” from the healthy phenomenon (e.g. inner speech) to the pathological phenomenon (i.e. AVH). I will then present three subtypes of AVH and show how the PPF accommodates different aetiological models for each.

### 5.1 Dissolving the Auditory Phenomenology Challenge

Viewed from within the PPF, this challenge is based on a mistaken understanding of how the brain works, and the relationship that this has to conscious experience.

The PPF changes how one thinks of perceptual experience, and, by extension, radically changes one’s explanatory focus in trying to account for hallucinations. On a standard framework, where front-line sensory stimuli get gradually processed and passed on up the hierarchy, hallucinations make one wonder, “Where does this erroneous sensory stimulus come from?” Indeed, we can see SMTs as making attempts to answer this within a standard framework. Their answer is: they come from the quasi-sensory stimulation of inner speech, which is then misattributed. However, when, instead, we adopt the PPF, incoming stimuli play a much smaller role in determining the conscious percept, even where veridical perception is concerned. Given that a conscious percept is constituted by the hypothesis that best minimises prediction error, we don’t ask, “Where does the input come from?”, since the input alone doesn’t (and can’t) determine the percept. Rather we ask, “Why does this hypothesis minimise prediction error?” This general approach makes hallucinations both less perplexing, and less different from veridical perception.^10^ The point is that on the PPF, your conscious experience at any given time is the hypothesis that your brain has selected in order to minimise prediction error. Applied to AVHs, your experience will have auditory phenomenology if your brain has had to adopt the hypothesis that you are hearing something in order to minimise prediction error.

This isn’t really a “transformation” because, on the PPF, it makes no sense to talk of inner speech as a “raw material” that needs transforming. Either you have an experience that is inner speech, because your brain has adopted the hypothesis that that is what is going on, or you have a different experience, corresponding to a different hypothesis. There is no *experience* of inner speech *first*, which is somehow then transformed. The question about whether inner speech is relevant for AVHs is not one of transformation. It is about whether relevant elements involved in the production of the inner speech experience are also involved in the production of some AVHs.^11^ It seems fairly clear, given all of the evidence in its support (some of which we have already seen, some of which we will touch upon below), that the answer to that is: yes.

However, it is worth noting that on the PPF, all action will be couched in terms of active inference, namely, in terms of (hopefully) self-fulfilling sensory predictions (cf. Adams et al., 2013). How we are to think of inner speech within this framework is a fascinating direction for future inquiry. Given the plausible view that inner speech is developmentally derived from overt, private, speech (Berk, 1992; Vygotsky, 1934; Winsler, 2004), this could involve activating a prediction (as for any overt action), but over time somehow managing to dispense with many of the features of the action in question. This could involve learning to lower the weighting on the prediction error, or to make the prediction itself less demanding, or a combination of the two. Either way, prediction error could be minimised without having to go through with all aspects of the action (or, rather, its developmental precursor, private speech), although some aspects (e.g. subvocalisations, muscular activations) will remain. How this could lead to AVH is something that we will come to shortly. The key idea, roughly, is that inner speech is stripped down outer speech, but that this is built back up in AVH. However, it is not simply built back to overt speech, but to something different that involves the hearing of speech, but not its production.

### 5.2 Accounting for three subtypes of AVH

Jones (2010) expressed a concern that one model is unlikely to explain the various subtypes of AVH. He presented two subtypes. I will add a third but then gesture towards how the PPF provides a framework against which all three can be explained through different aetiological models.

#### 5.2.1 Inner speech hallucinations

As we mentioned, the most popular model for understanding AVHs involves misattributed inner speech, where the misattribution results from an abnormality in self-monitoring. Although I want to abandon the framework within which SMTs tend to function, following Jones (2010), I think it is important to emphasise that there is great deal of empirical support for the view that a subset of AVHs implicate mechanisms involved in inner speech.

There is support for the idea that inner speech might be relevant, other than the experiments we noted earlier in support of SMTs. Ford and Mathalon (2004) used EEG findings to suggest that during the production of healthy inner speech, corollary signals from speech production areas seem to suppress activation in speech processing areas. As Bentall and Varese (2013) nicely put it, “it might be said that when we talk to ourselves, the frontal language production areas of the brain tell the posterior areas not to bother listening” (2013, p.71). This attenuation should remind us of the support from bodily effects of monitoring, namely, the attenuation of sensation in (attempted) self-tickling. Both the attenuation pertaining to self-tickling and to inner speech can be accounted for within the PPF.

Within the PPF, the fact that we cannot tickle ourselves is explained rather differently from how it is explained in SMTs that rely on motor commands and self-monitoring mechanisms. Brown and et al. (2013) have done work within the PPF that accounts for this in terms of a more general dampening of weighting on prediction error. The reason for this dampening is that movement involves implementing a currently false hypothesis (“I am moving”) in favour of the currently true hypothesis (“I am not moving”). The dampening swings the balance in favour of the false hypothesis, which then becomes true when the action is initiated.^12^ In inner speech, there is the prediction that you will speak, but the precision weighting will be turned right down, circumventing the need to minimise prediction error by actually overtly speaking (although, as we saw, some vestiges of the overt speech will remain). This will correspond to the suppression of activation in “speech processing areas” which are reported in both inner speech and in hearing oneself speak aloud (Ford & Mathalon, 2004). On the PPF there would be less activation in these areas, even when hearing oneself speak aloud, since there would be less prediction error (better predictions) than hearing someone else speak.

The fundamental principle of prediction-error minimisation is the same whether the activity to be predicted is self-generated or not. One important difference, however, is that, if the activity is self-generated, the expected precision will be higher. Given this, only a relatively slight error in prediction would result in highly weighted prediction error, which would subsequently result in a more erroneous hypothesis to explain it away. This could account for cases where there are delusions of control and inner speech-based hallucinations in the absence of more outwardly directed perceptual abnormalities.

#### 5.2.2 Memory-based hallucinations

Waters et al. (2006) distinguish their view from “standard cognitive accounts”, by which they mean SMTs, and which they call “single deficit accounts”, by proposing that:

at least two cognitive deficits must be present to explain auditory hallucinations: (1) a fundamental deficit in intentional inhibition which leads to auditory mental representations intruding into consciousness in a manner that is beyond the control of the sufferer; and (2) a deficit in binding contextual cues, resulting in an inability to form a complete representation of the origins of mental events.

[Waters et al. (2006, p.66)]

This talk of “auditory mental representations”, “inhibition” and “binding of contextual cues” is nicely illustrative of how removed this is from the sort of framework that I am suggesting in this paper. However, this is not to say that they are incompatible, and cannot both be helpful.

Using a couple of tasks (the Hayling Sentence Completion Task (Burgess & Shallice, 1996) and the Inhibition of Currently Irrelevant Memories Task (Schnider & Ptak, 1999)) Badcock, Waters, Maybery, and Michie (2005) demonstrated that hallucinating patients with diagnoses of schizophrenia have lower inhibition (more intrusion) of irrelevant associations and memories. The second deficit is needed to explain why these memories, which are failing to be inhibited, aren’t being recognised as memories. In research on episodic memory, there is a distinction between content (what is remembered) and context (e.g. temporal and “source” features of what is remembered). Waters et al. (2006) suggest that, although the content is remembered, there is a deficit in “context memory”, and, more specifically, “an impairment in combining contextual cues together to form an integrated representation of an event in memory”. Thus, the memory will present something that happened in the past, usually something traumatic, but it will not be experienced as a memory. It will be taken as a voice from the present.

Let us look at what might be going on here through the lens of the PPF. In a case of an episode of healthy episodic memory, whether deliberate or unbidden, what is involved is the activation of relevant imagery, which within the PPF (in a way somewhat similar to our earlier discussion of inner speech) is not some kind of inner sensation, but a prediction. However, unlike the predictions at play in perception and action, this prediction is not answerable to – malleable in the light of – inputs. How is this “decoupling” from the environment achieved? What needs to happen is that the prediction need not to be “taken too seriously”, and so this will mean turning the precision right down on prediction error (this is similar to what happens in inner speech, minus the articulatory component). That way the “prediction” can be maintained even though the system is fully aware that it is not really predicting anything, that there is nothing really corresponding to the localised hypothesis generating the prediction. The general, overarching, hypothesis, is still that the subject is where she is (e.g. at a desk), but she is simply remembering something (e.g. that time when her father shouted at her).

Now suppose that this prediction is activated, but there is a problem with keeping the weighting on prediction error low. This generates erroneous amounts of prediction error for which the brain has to adopt a hypothesis: this is perception, not memory. In a sense, then, if predictive processing, and, in particular the upwards and downwards modulation of precision on prediction error, goes badly awry then we get a blurring between mere imagery (as involved in episodic memory, imagination and, with relevant qualification, inner speech) and perception. The clever trick of decoupling, which enables us to remember episodically, or to imagine vividly, is disrupted.

However, if this is correct, then we don’t need to appeal to integration of contextual cues (except insofar as there is a *de facto* failure to realise that this is not happening now). The recollective episode is not recognised by the subject as a memory, not because of some cognitive failure to remember the temporal or “source” context in which this happened, but because it doesn’t feel phenomenologically like remembering something: it feels like hearing something. Indeed, failure to remember temporal or source context cannot suffice to explain AVHs since there are cases where the voices are recognised by the subject as being exact replays of the past (12% in McCarthy-Jones et al., 2012), but they are still not experienced as *memories,* but as perceptual experiences.

Indeed Wu’s *Auditory Phenomenology Challenge* applies to memory-based context-monitoring views almost as much as to inner-speech-based self-monitoring views. I say “almost as much” since perhaps (and we know very little about whether this is actually the case) episodic auditory memories have more phenomenological features in common with auditory perceptual experiences than episodes of inner speech (which arguably have an active, articulatory component). But, in any case, they certainly do not share all of them. So the challenge goes: How does one account for the auditory phenomenology of AVH if they are simply mis-identified memories? How does one account for the “transformation” from auditory-memory phenomenology to auditory-experience phenomenology? On the PPF account, since the conscious experience is a product of prediction-error minimisation, this question doesn’t arise. The initial event, the explanatorily relevant genesis of the experience, may be (as with the inner speech hallucinations) the same initial event as for a recollection in episodic memory, however, the erroneous prediction error causes the brain to hypothesise that something very different is going on.

#### 5.2.3 Hypervigilance Hallucinations

The two varieties I have just elaborated on were introduced earlier in the paper through a consideration of Jones’s *Varieties Challenge*. This third kind has not yet been presented in this paper.

Perhaps the first step towards an appreciation of the existence of hypervigilance hallucinations was made in a paper by Delespaul, DeVries, and Van Os (2002), who conducted an investigation into the contextual influences on AVHs. They found that the context where voice hearing is most likely to occur is either in the presence lots of people or alone. Building on this, Dodgson and Gordon (2009) suggested, on grounds of clinical case-studies, a kind of AVH called a “hypervigilance hallucination.” Here is a description of hypervigilance hallucinations from their paper:

Michael was experiencing a series of stressors including break up with a girlfriend and exclusion from seeing their child, heavy street drug use, including amphetamine, and a pending court case for arson. He began to experience auditory hallucinations, hearing people calling him a “nonce”. Michael began to believe that people could read his thoughts, and that people thought he was a paedophile.

[Dodgson and Gordon (2009, p.328)]

The existence of hypervigilance hallucinations as a separate subtype was subsequently supported by Garwood, Dodgson, Bruce, and McCarthy-Jones (2013) who, based on a cluster analysis, showed that AVHs tend to occur when:

(i) attention is directed *inward* in *quiet* contexts

(ii) attention is directed *outward* in *noisy* contexts

They took (i) to suggest that inner speech hallucinations were occurring, and (ii) to suggest that hypervigilance hallucinations were occurring. Attention seems like a key factor here. So let’s view this notion of hypervigilance from within the PPF and its gloss on attention as precision-weighting.

Let us contrast vigilance, with an illustratively opposing state: total calmness and lack of threat. Compare two cases of walking down a familiar woodland path at dusk. In the one case, you are walking home after an agreeable dinner party at a friend’s house, and you are feeling happy and relaxed. Because it is getting dark, you will find that your brain is forming rather vague hypotheses about what is out there, but since you are relaxed, and you have no reason to think that anything might be a threat, this is fine: precision-weighting (“attention”) is kept low. You get home safely having represented your environment in a vaguer, more coarse-grained way than you would have done in broad daylight, but this served your navigational purposes. Now suppose, instead, that you are walking home after having watched a horror movie. You are no longer relaxed, but in an emotional state of *vigilance*. As a result, you will be more likely to interpret something as looking like a person lurking amongst the trees. If this startles you, your attention will focus on that part of your visual field, and the precision-weighting of prediction error in that area will be turned right up. You may, due to this upwards-modulation, see that it is, in fact, only a tree trunk. Although as you look more closely, you get more incoming visual information suggesting the tree-trunk hypothesis, *interoceptive* information, namely, the fear that you feel, which is residual from the horror movie you saw, counts in favour of the lurking-person hypothesis (Pezzulo, 2013). You (or, perhaps more accurately, your nervous system) roughly think: “Why am I scared? There must be something to be scared of”, and then you get even more scared. In sharp contrast, in the first case, when you are happy and relaxed, the lurking-person hypothesis doesn’t even present itself.

This fits nicely with hypervigilance hallucinations. In a state of hypervigilance, ambiguous inputs will be given a threatening interpretation; hypothesis selection will be biased towards something threatening, because this will also serve the purpose of explaining the *interoceptive* state (Seth, 2013). For the subject, this will give rise to experiences which misrepresent reality: from the ticking clock or the muffled sound of neighbours talking will emerge the experience of a voice telling the subject exactly what the subject, in his state of hypervigilance, is afraid of hearing (e.g. “nonce!”).

Unlike the two other subtypes, hypervigilance hallucination have their genesis in the external world. One might of course claim that, since hypervigilance hallucinations do, strictly speaking, have some kind of worldly stimulus, then they are not, *strictly speaking*, hallucinations; they are rather illusions. I see this as a mainly terminological point, however, so will not go into it.^13^ A more important point is that, because of this, SMTs cannot account for hypervigilance hallucinations whereas the PPF can. The point is that, according to SMTs, normal monitoring exploits mechanisms (e.g. motor commands) linked to the production of self-generated events, and it is this that goes wrong and is misattributed. Thus, although it has a story to tell about how bodily actions, inner speech, and perhaps even episodic memories, can be badly monitored, and hence misattributed, it must remain silent about hypervigilance hallucinations, which are precisely not a case of a self-generated stimulus being misattributed, but rather an external stimulus being misinterpreted. To put it another way, unlike with SMTs, in the PPF, *all* stimuli, not just the self-generated ones, need to be predicted.

### 5.3 Multiple models within one framework: personal history and attentional focus

I have gestured towards how these three subtypes could be accounted for within the PPF, but why would a given subject, at a given time, experience one subtype rather than another? This is an unavoidably difficult question. Although I cannot answer it in full, I think that two things are of particular explanatory relevance within the PPF. The first is the personal history of the subject; the second is the subject’s attentional focus, which will probably often be the accompaniment of certain moods, emotions and contexts.

Personal history, for example, whether a subject underwent some form of traumatic abuse as a child, is likely to have an impact on whether a subject hears voices, and, as we have suggested, these voices are perhaps likely to be memory-based. Similarly, if a subject (like Michael, in Dodgson and Gordon’s case-study) has a history of amphetamine use, coupled with other so-called “stressors”, this might well lead to heightened risk of AVH, but probably not memory-based ones, since there is no single traumatic event to form the basis of the relevant episodic memory. Of course there could be interaction between the different factors that contribute to the (perhaps unrealistically clear) delineations of the subtypes. Thus childhood trauma and substance abuse could contribute to someone being at heightened risk of AVH. These might be, for example, hypervigilance hallucinations whose content could in some way be tied to past trauma.

What, then, would make an individual more likely to experience AVHs that have an internal (viz. inner speech or memory) or an external (viz. hypervigilance) genesis? In answer to this question it may be useful to appeal to attentional focus. We saw that, within the PPF, following Hohwy (2012), we can think of attention as turning up the precision-weighting, and hence potential prediction error, on the attended stimulus. Attention can be directed outward, at incoming stimuli, or it can be directed inward, at thoughts, feelings and memories. Thus, if attention is directed outward in a state of anxious hypervigilance, there is more likely to be excessively weighted prediction error from the outside. This would correspond to hypervigilance hallucinations. As a result, traffic noise, or a ticking clock, or the sound of a group of people talking, might be embellished into the experience of a voice saying something. Alternatively, if attention is directed inwards, and the subject is socially isolated, ruminating in a state of shame or guilt, then there is more likely to be excessively weighted prediction error from the inside. This would correspond to inner speech or memory-based hallucinations. This nicely fits the findings by Garwood et al. (2013), concerning the prevalence of hallucinations in quiet and noisy contexts. It also allows that one patient, in different contexts and different moods, may experience different AVH subtypes. Indeed all three of the subtypes presented here may occur in one subject at different times. Of course, the picture is more complicated, but the framework is precisely the sort that is capable of accommodating such complications on a case-by-case basis.

## 6 Summary and conclusion

I have suggested that we view AVHs through the lens of a predictive processing framework as a way of addressing two challenges, the first of which, in particular, plagues orthodox SMTs, the second of which is problematic for any single model of AVHs.

The first challenge, of how the auditory phenomenology of AVHs can be accounted for, or at least rendered less perplexing, is addressed by the fact that the subject’s conscious percept is not determined by incoming stimuli, but by the brain’s best hypothesis as selected on the basis of how well it minimises prediction error. The question is not: Where does this erroneous stimulus come from? Rather the question is: Why does the brain adopt such a strange hypothesis? The answer to this question is (at least partially): erroneous precision weighting on prediction error.

The second challenge, concerning the varieties of AVH, is addressed by realising that a new framework can accommodate elements of pre-existing models, but views the contribution of, for example, episodic memory or inner speech, in a way that is somewhat different. They are not “raw materials” to be transformed. Rather, mechanisms that are implicated in generating healthy experiences of episodic memory and inner speech, can generate erroneous prediction error that will result in erroneous hypotheses being adopted to minimise it. A major advancement of the PPF over SMTs is the realisation that, since prediction lies at the heart of cognition (broadly construed to include emotion and action), both internally and externally produced stimuli need to be predicted. SMTs had to be silent regarding external stimuli, since only self-generated stimuli can be monitored.

Adopting the PPF, like adopting any framework, is only a first step. Not only is there, first of all, work to be done on better understanding predictive processing; for example, its neurobiological underpinnings and how it might go wrong at that level (see Corlett et al., 2010 for advancements in this direction). There is also exciting work to be done within the PPF with specific case-studies. It is a flexible framework within which we can better understand unique individuals, with different histories, problems and conditions.^14^

## Footnotes

1 Later I present a framework that does away with motor commands as traditionally construed, even for overt bodily actions.

2 One can also question the claim that AVHs always have auditory phenomenology. For example, Frith (1992, p.73) maintains that AVHs can involve “an experience of receiving a communication without any sensory component”.

3 As Jones puts it, “[A]lthough models are created to fit the phenomenology of AVHs, due to the diverse nature of the experience they inevitably end up only fitting specific types of AVHs. Whilst it is possible a grand unified theory of AVHs and AHs may be developed, possibly involving a neurological mechanism cutting across many cognitive, metacognitive, linguistic and/or perceptual capacities, at present no such model exists” (Jones, 2010, p. 573).

4 Although, of course, the adoption of frameworks will tend to be motivated by theoretical commitments. For example, the PPF is motivated by theoretical commitments concerning brain function. But this is a framework because it provides a way of approaching to a wide variety of issues.

5 One might say what is “out there”, but a great deal of information is internal to the body (see Seth (2013) on interoceptive predictive processing).

6 To quote Hohwy’s rather stronger claim (and one that I would not assent to without serious qualification): “prediction error minimisation is all the brain ever does” (2013, p.7).

7 Theorists tend to take the mechanism of precision-weighting to involve specifically the prediction error rather than the predictions themselves. That is to say, the prediction error is given more weight in high-precision contexts. This amounts to the system being more demanding in terms of precision. However, the same effect could be achieved, not at the level of prediction error, but at the level of the precision of the priors (i.e. the predictions themselves). If the priors are more precise, then you are likely to get more prediction error. Distinguishing between these will not amount to a computational difference. It will (depending on how robustly realist one is about the PPF) either be an implementational difference, or simply to two ways of talking about the same thing. Either way, in contexts of high-precision, you will get either more prediction error, or prediction error that carries more “weight”.

8 Similarly, playing loud audio recordings of crowded rooms was used as a simple counter-surveillance technique. Two people can easily converse by looking at each-other’s mouths producing the sounds. Their brain uses predictions generated by the visual information to pick out the relevant auditory information from the noisy background. Anyone listening in on a bug will lack the necessary visual cues to pick out the conversation from the noise in the same way.

9 There’s obviously a similar distinction to be drawn between your expectations (say, when placing a bet) and your brain’s priors.

10 In some respects, Helmholtz was quite right when he said that “Perception is controlled Hallucination”. This is trivially false if “hallucination” is understood normatively, as being *by definition* inaccurate: Helmholtz meant this to apply to fully veridical perceptual experience. What he meant was that, to put it bluntly, the vast majority of the “raw materials” for our perceptual experiences are prediction that originate in our heads, with an important, but informationally tiny role, given to incoming signals.

11 This resembles what Wu (2012) calls “the moderate identification thesis”, which he claims to not be taking issue with. The strong identification thesis, which he criticises on empirical grounds, I would criticise on conceptual grounds. It’s not *even* false: it’s confused. You can’t say that AVHs just are episodes of inner speech. One experience cannot be the same as a different experience!

12 So both PPF and SMTs have an account of why we cannot tickle ourselves, but is there anything to adjudicate between them? Well, they do generate different experimental predictions. SMTs view ticklishness as a function of unpredictability of specific movements that would be rendered predictable through self-monitoring. PPF on the other hand, views it as a more general dampening of precision on prediction error.

13 One can still be justified in calling them hallucinations. The distinction between hallucination and illusion is not necessarily one between two different loci of initial stimuli, but between two different experiences and how they represent relevant aspects of the world. In the one, something is represented which isn’t there at all. In the other, something is presented but it is distorted. Viewed in this way, the experience in hypervigilance hallucinations is unrecognizable from the causal source of the stimulus. Something that is totally absent is being presented to the subject, regardless of whether something actual in the world is causally implicated. That cause is no longer in any way represented in the content of the experience.

14 I’d like the mention the possibility that the PPF might be useful for understanding voice hearing in other disorders (e.g. in major depression, bipolar disorder or PTSD) (Larøi et al., 2012), and, indeed, in the non-clinical population (Beavan, Read, & Cartwright, 2011; Larøi, 2012).
